# P05.54. Thermometer of warmth in the patient-provider relationship (WARMOMETER) - a short and easy to understand measure for use in integrative medicine

**DOI:** 10.1186/1472-6882-12-S1-P414

**Published:** 2012-06-12

**Authors:** M Neumann, C Scheffer, D Cysarz, D Tauschel, L Taylor-Swanson, F Edelhäuser

**Affiliations:** 1 University of Witten/Herdecke, School of Medicine, ICURAM, Herdecke, Germany; 2 University of Washington, School of Nursing, Seattle, USA

## Purpose

The aims of this study are twofold: (1) the theory-based development of a patient self-report measure of physician warmth; and (2) the application of cognitive interview methodology to understand patients’ perception and interpretation of this new measure.

## Methods

A draft measure was developed based on an in-depth literature review of the concept of human warmth by a multidisciplinary expert group working in integrative medicine. Sixteen cognitive probing interviews were conducted to examine how patients from integrative and conventional medicine perceive and interpret this new measure and to identify potential problems. A content analysis of the interviews was used to evaluate findings.

## Results

Findings indicate that the WARMOMETER is a short patient self-report assessment of physician warmth, which seems easy and intuitive to understand for every patient. In addition, most respondents were found to share a common concept of physician warmth.

## Conclusion

Verification of our study hypotheses and confirmation of the theoretical assumptions of human warmth give basic indications that the WARMOMETER seems to be a valid and sensitive patient self-report instrument for assessing the socio-emotional quality of physicians in integrative and conventional medicine. These first promising results of our cognitive interviews suggest that the WARMOMETER may also be used and further validated in future studies on the patient-physician relationship in integrative medicine, also with other healthcare professionals, e.g. nurses.

